# Assessment of paralogue annotation for improving diagnostic accuracy in *CALM1*, *CALM2*, and *CALM3* genes

**DOI:** 10.3389/fgene.2026.1761341

**Published:** 2026-07-15

**Authors:** Kathryn M. Curry, Amar Mujkic, Natalie Syverud, Anna K. McGill, Jane E. Beckwell, Elizabeth A. Wiley, Jeffrey Bissonnette, Amina Kurtovic-Kozaric, Mark J. Kiel

**Affiliations:** Genomenon, Ann Arbor, MI, United States

**Keywords:** CALM1, CALM2, CALM3, calmodulin, paralogue annotation

## Abstract

The calcium (Ca^2+^) sensor calmodulin (CaM) genes *CALM1*, *CALM2*, and *CALM3* were recently included in the American College Medical Genetics and Genomics (ACMG) secondary findings (SF) list, given their significance in causing long QT syndrome (LQTS) and catecholaminergic polymorphic ventricular tachycardia (CPVT). These three genes share identical protein sequences, posing potential challenges in variant interpretation. Using paralogue annotation (PA) to classify pathogenic variants and variants of uncertain significance (VUS) across these three paralogue genes, we performed a systematic, semi-automated curation of the *CALM1*, *CALM2*, and *CALM3* variants. The analysis identified 173 unique CALM variants from ClinVar and Mastermind databases (75 *CALM1*, 59 *CALM2*, and 39 *CALM3* variants). After paralogue annotation, we identified 126 unique variants in each of the three genes—378 cDNA variants in total. Out of 126 unique variants for each CALM gene, 63 were VUS, 62 were likely pathogenic/pathogenic (LP/P), and one was conflicting (192 VUS, 186 P/LP, and 3 C in total). Twelve unique variants in the *CALM1*, *CALM2*, or *CALM3* genes had conflicting classifications between VUS and LP/P calls, which were resolved as LP/P. The application of paralogue annotation and variant curation resulted in an increased number of likely pathogenic/pathogenic variants (111% increase). Additionally, our analysis confirms that the majority of known pathogenic variants are predominantly located within the C-lobe of the CaM protein. This study highlights the benefits of paralogue annotation for accurate variant interpretation in *CALM1*, *CALM2*, and *CALM3* genes, suggesting that a reduction in missed diagnoses is associated with calmodulinopathies.

## Introduction

The American College Medical Genetics and Genomics (ACMG) has proposed a list of genes that clinical laboratories should report that are distinct from the primary reason for conducting the sequencing, termed “secondary findings” or “SF” genes (Green et al., 2013). At the time of writing, the most recent report on secondary findings included *CALM1*, *CALM2*, and *CALM3* genes for their associations with long QT syndrome (LQTS) types 14–16 (Miller et al., 2023). All three of the CALM genes code for the same protein, calcium (Ca^2+^) sensor calmodulin (CaM), which demonstrates remarkable conservation across species (Jensen et al., 2018). Multiple sequence alignment of the translated transcripts of the genes shows that they are identical in amino acid composition, with some cDNA-level differences (Supplementary Figures S1, S2). The CALM genes do differ in evolutionary constraint, expression levels, and contribution to the total calmodulin pool, with *CALM1* and *CALM2* accounting for the majority of cardiac calmodulin production and *CALM3* contributing substantially less (Christiansen et al., 2025).

The first identified pathogenic variant in the *CALM1* gene was discovered by Nyegaard et al. (2012). Since then, *CALM1*, *CALM2*, and *CALM3* have been classified as “definitive” for LQTS, with evidence from families with the syndrome (Crotti et al., 2013; Pipilas et al., 2016). In addition to LQTS, which has been noted in publications regarding idiopathic ventricular fibrillation with mild QTc prolongation (Marsman et al., 2014), CALM genes are also associated with catecholaminergic polymorphic ventricular tachycardia (CPVT) (Nyegaard et al., 2012; Gomez-Hurtado et al., 2016; Gao et al., 2023). It has been proposed that the CALM genes may also be associated with primary neurological/neurodevelopmental features in cardiac patients, but evidence is currently limited as to whether a primary, isolated neuro presentation of the disease is a separate true gene-disease association (Crotti et al., 2023). Nevertheless, many CALM gene variants that have been reported in the literature or in online databases remain variants of uncertain significance (VUS). Considering that these genes are reported separately in online databases and in NGS analysis, the same pathogenic variant in one CALM gene may not be automatically recognized as potentially pathogenic in another. Given the potential severity of the associated diseases, misdiagnosis could have fatal consequences and missed opportunities for cascade genetic testing.

Since all three genes encode the same protein with cDNA differences leading to aligned amino acids across the genes, we applied the hypothetical application of paralogue annotation (PA), which allows us to infer baseline knowledge of a coding region missense variant found in one of the genes to another gene. We asked, “If a variant is known to be causative in one CALM gene, what does it mean when we observe a variant at the same aligned residue in its paralog?” This study employs three different methods to advance variant curation of the three CALM gene variants.

1. The identification of variants associated with the *CALM1*, *CALM2*, and *CALM3* genes from the ClinVar database.
2. An exhaustive literature examination employing the Mastermind Genomic Search Engine to gather supplementary variants and corroborating evidence.
3. Hypothetical paralogue annotation application on all the collected variants from both ClinVar and Mastermind literature-derived variants.

Through these approaches, we identified all reported variants found among the *CALM1*, *CALM2*, and *CALM3* genes and applied the paralogue annotation concept to determine the risk of and reduce the potential for further misdiagnosis.

## Materials and methods

### Data extraction

**FIGURE 1:**
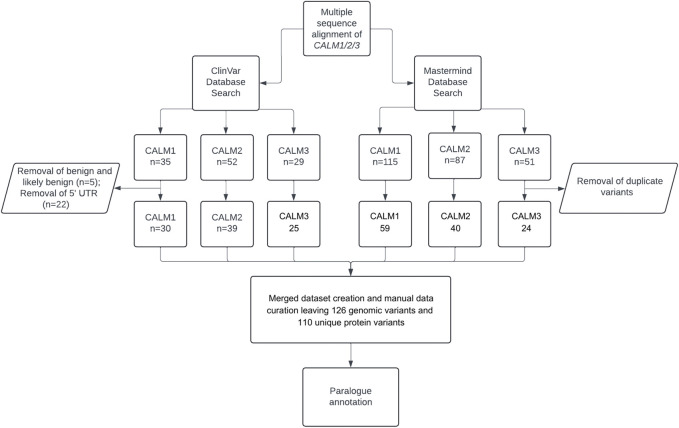
Methodological approach for paralogue annotation (PA) of *CALM1*, *CALM2*, and *CALM3* variants. ClinVar and Mastermind databases were used to identify *CALM1*, *CALM2*, and *CALM3* missense variants. Non-missense, duplicate, benign, and likely benign variants were removed. The two datasets were merged and manually curated according to ACMG guidelines based on evidence available for each individual gene prior to PA. The final dataset comprised 126 unique cDNA or 110 protein variants.

The analysis involved the four steps shown in Figure 1: 1) extraction of variant data from Mastermind (Mastermind, Genomenon) and ClinVar databases for pathogenic/likely pathogenic missense variants in *CALM1*, *CALM2*, and *CALM3* genes (Supplementary Table S1); 2) expert curation of collected variants according to the ACMG/Association for Molecular Pathology (AMP) criteria (Richards et al., 2015), referred to as “ACMG criteria” hereafter, for gene–disease associations with at least moderate level of evidence: CPVT and LQTS; 3) assembly of curated variants into one database; and 4) paralogue annotation (Supplementary Table S1).

Variant nomenclature was normalized according to the UniProt-associated RefSeq canonical transcripts: *CALM1* (NM_006888.6), *CALM2* (NM_001743.6), and *CALM3* (NM_005184.4). Multiple sequence alignment on all three translated sequences was performed using Clustal Omega (Supplementary Figure S1). ClinVar data was downloaded on 17 April 2024 with the “missense” and “single-nucleotide variant” filters applied for *CALM1*, *CALM2*, and *CALM3*. All three entries for these genes were downloaded and combined into a single file (Figure 1; Supplementary Table S1). Benign, likely benign, and 5’ UTR variants were removed. dbscSNV and SpliceAI scores were calculated for all variants (Supplementary Table S2) (Jian et al., 2014; Jaganathan et al., 2019). A Mastermind Genomic Intelligence Platform-based literature search identified variants from published journal articles containing at least one variant in the CALM genes. Mastermind is a genomic evidence search engine that organizes genetic variant data from the published literature and associated datasets to facilitate the finding correlations between different gene variants and particular traits or diseases (Chunn et al., 2020).

### Data reconciliation and paralogue annotation

After the data extraction and cleanup of duplicates and benign variants, *CALM1*, *CALM2*, and *CALM3* variants from Mastermind were curated according to the ACMG criteria (see Supplementary Table S3 for Mastermind’s classification standards and definitions of ACMG codes). In brief, evidence for each variant was systematically collected through literature curation and the integration of population databases (e.g., gnomAD), computational prediction tools, and gene- or disease-specific contextual factors. Variants were evaluated across multiple evidence categories, including population frequency, computational predictions, intrinsic variant features, clinical observations, and functional studies, with criteria applied at defined strength levels. Table 2 and Supplementary Table S3 contain definitions of the ACMG criteria applied. Conflicting or insufficient evidence was handled through specific classification categories (e.g., “conflict” or VUS), and all classifications are considered provisional and subject to revision as new data emerge. The final classification was determined by combining weighted evidence according to ACMG/AMP rules, with transparency and traceability to supporting evidence maintained throughout the process.

In regard to gene–disease associations for CALM genes, patients can contribute as a proband with gene–disease-associated phenotypes that fall within the LQTS and CPVT definitions as supported by ClinGen’s LQTS and CPVT Gene Curation Expert Panels (GCEPs), the associations of which align with our internal gene–disease relationship analysis (Adler et al., 2020; Walsh et al., 2022). Patients cannot contribute to pathogenicity criteria if they were reported to have been presented with other limited-association phenotypes.

Once the curation process was complete and a classification call was assigned to each variant, evidence from the Mastermind and ClinVar databases was compiled into a single dataset. Both likely pathogenic (LP) and pathogenic (P) variants were labeled as “P” variants and are treated as such throughout this analysis. Variants with the same cDNA change within one gene that had a discrepant call between databases, such as P/LP vs. VUS, underwent an additional assessment to reach a final suggested classification. This assessment involved a detailed review of ClinVar entries for any missing literature-derived evidence and reassessment of all previously curated data according to ACMG standards.

After the data were merged and discrepant calls reassessed, a single database of *CALM1*, *CALM2*, and *CALM3* variants was created (Supplementary Table S1). The paralogue annotation (PA) concept was applied to the dataset, which assumes that if a variant was classified as P in one gene, the paralogous variants in the other two CALM genes with similar computational, *in silico*, and population frequency criteria are also hypothesized to be P/LP. Thus, for variants which did not have a reported classification from either dataset (unpublished variants), the suggested PA application code was applied to existing ACMG codes for the unpublished variant, with the suggested ACMG call provided in Table 2. For variants that had discrepant calls across CALM genes—if the variant was classified as VUS in *CALM1* and P in *CALM2*—the final classification was P for all three CALM genes as the addition of the strong PA application code increases the pathogenicity score of the variant.

### Variant mapping

The calmodulin (CaM) protein was modeled computationally using the PyMOL Molecular Graphics System (DeLano, 2002). This model functioned as a virtual platform to illustrate how the pathogenic variations were positioned on the protein. Ca^2+^ bound CaM in the holo-form, 1CLL, was retrieved from the Protein Data Bank (PDB) for computational modeling (Chattopadhyaya et al., 1992). Hydrogen atoms and ethanol molecules were removed from the protein before analysis.

## Results

### *CALM1*, *CALM2*, and *CALM3* variants from ClinVar

**FIGURE 2:**
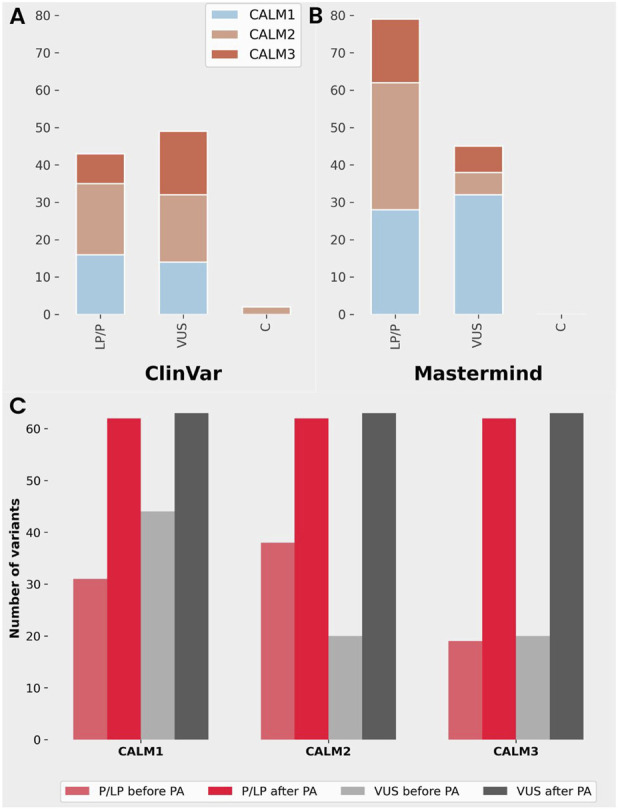
Number of pathogenic and VUS variants in *CALM1*, *CALM2*, and *CALM3* genes in ClinVar **(A)** and Mastermind **(B)**. Abbreviations: VUS, variants of uncertain significance; P, pathogenic variants reported as either pathogenic or likely pathogenic. **(C)** Conflicting classification of pathogenicity. Application of paralogue annotation (PA) to *CALM1*, *CALM2*, and *CALM3* genes. Before and after PA, the use of PA led to a 111% increase in P across *CALM1*, *CALM2*, and *CALM3* genes.

The ClinVar dataset comprised 94 cDNA variants (70 unique protein variants) after the removal of duplicates, benign variants, and variants from the 5’ UTR region, comprising 30 *CALM1* variants, 39 *CALM2* variants, and 25 *CALM3* variants (Table 1). Out of 94 cDNA variants, 49 were VUS, four were classified as P/LP, 16 were LP, 23 were pathogenic P, and two variants had conflicting pathogenicity classifications (Figure 2A; Table 2).

**TABLE 1:** Comparison between total number of variants and conflicting classification calls between the ClinVar and Mastermind databases. The pathogenic, VUS, and conflicting calls were counted and compared between ClinVar and Mastermind for *CALM1*, *CALM2*, and *CALM3* genes before paralogue annotation.

ACMG call	CALM1	CALM2	CALM3	Total
ClinVar				
Likely pathogenic/pathogenic	16	19	8	43
VUS	14	18	17	49
Conflict	0	2	0	2
Total	30	39	25	94
Mastermind				
Likely pathogenic/pathogenic	27	34	17	78
VUS	32	6	7	45
Total	59	40	24	123
Classification overlap in ClinVar and mastermind				
Pathogenic in both databases	12	15	6	33
VUS in both databases	0	1	0	1
Classification disagreement ClinVar and mastermind				
VUS (ClinVar) vs. LP/P (mastermind)	1	2	3	6
LP/P (ClinVar) vs. VUS (mastermind)	1	1	1	3
Conflict (ClinVar) vs. LP/P (mastermind)	0	1	0	1

**TABLE 2:** Raw data before and after PA. First three columns are cDNA change, protein variant, and domain of the variants assessed. Columns D–F are *CALM1*, *CALM2*, and *CALM3* variants from ClinVar. Columns G, H, and I 4–6 are variants reported in the literature present in Mastermind. Columns J–L are ACMG criteria applied to the Mastermind variants. Columns M–O are the merged classification between the two datasets (ClinVar and MasterMind). Lastly the consensus columns represent the final ACMG classification for variants across *CALM1*, *CALM2*, and *CALM3* genes after paralogue annotation.

​	​	​	ClinVar			Mastermind			Applied criteria			Merged classification			Paralogue annotation code application hypothesis consensus P/LP calls lumped as (P)		
cDNA change	Protein variant	Domain	*CALM1*	*CALM2*	*CALM3*	*CALM1*	*CALM2*	*CALM3*	*CALM1*	*CALM2*	*CALM3*	*CALM1*	*CALM2*	*CALM3*	*CALM1*	*CALM2*	*CALM3*
c.8G>A	R3H	N/A	​	VUS	​	​	​	​	​	​	​	​	VUS	​	VUS	VUS	VUS
N/A	Q9A	EF-hand 1	​	​	​	VUS	​	​	fx|pm2|pp2	​	​	VUS	​	​	VUS	VUS	VUS
c.29T>C	I10T	EF-hand 1	​	​	​	​	VUS	​	​	fx|pm2|pp2|pp3	​	​	VUS	​	VUS	VUS	VUS
N/A	F13M	EF-hand 1	​	​	​	VUS	​	​	fx|pm2|pp2	​	​	VUS	​	​	VUS	VUS	VUS
c.44A>C	E15A	EF-hand 1	​	​	​	VUS	​	​	fx|pm2|pp2|pp3	​	​	VUS	​	​	VUS	VUS	VUS
c.77G>A	G26D	EF-hand 1	​	​	​	VUS	​	​	fx|pm2|pp2|pp3	​	​	VUS	​	​	VUS	VUS	VUS
c.79A>T	T27S	EF-hand 1	​	​	​	VUS	​	​	fx|pm2|pp2	​	​	VUS	​	​	VUS	VUS	VUS
c.80C>G	T27S	EF-hand 1	​	​	​	VUS	​	​	fx|pm2|pp2	​	​	VUS	​	​	VUS	VUS	VUS
c.82A>G	I28V	EF-hand 1	​	​	​	VUS	​	​	fx|pm2|pp2	​	​	VUS	​	​	VUS	VUS	VUS
c.85A>T	T29S	EF-hand 1	​	​	​	​	​	VUS	​	​	pm2|pp2	​	​	VUS	VUS	VUS	VUS
c.86C>G	T29S	EF-hand 1	​	​	​	​	​	VUS	​	​	pm2|pp2	​	​	VUS	VUS	VUS	VUS
c.89C>G	T30R/S	EF-hand 1	​	​	​	​	LP	​	​	pm2|pp1|pp2|pp3|ppc	​	​	LP	​	P*	P	P*
c.104C>T	T35I	EF-hand 1	​	​	​	​	LP	​	​	pm2|pp1|pp2|pp3|ppc	​	​	LP	​	P*	P	P*
N/A	M36T	EF-hand 1	​	​	​	VUS	​	​	pm2|pp2	​	​	VUS	​	​	VUS	VUS	VUS
c.130C>T	P44S	EF-hand 2	​	VUS	​	​	​	​	​	​	​	​	VUS	​	VUS	VUS	VUS
c.131C>T	P44L	EF-hand 2	​	​	VUS	​	​	​	​	​	​	​	​	VUS	VUS	VUS	VUS
c.136G>A	E46K	EF-hand 2	​	VUS	​	VUS	LP	​	pm2|pp2|fx	pm2|pm6|pp2|ps4m	​	VUS	LP	​	P*	P	P*
c.143A>G	E48G	EF-hand 2	​	​	​	VUS	​	​	fx|pm2|pp2|pp3	​	​	VUS	​	​	VUS	VUS	VUS
c.149A>G	Q50R	EF-hand 2	​	​	​	VUS	​	​	fx|pm2|pp2|pp3	​	​	VUS	​	​	VUS	VUS	VUS
c.157A>G	I53V	EF-hand 2	VUS	VUS	​	​	​	​	​	​	​	VUS	VUS	​	VUS	VUS	VUS
c.161A>T	N54I	EF-hand 2	LP	​	​	P	LP	VUS	fx|pm2|pp3|pp1m|pp2|ppc|ppc_het|ps3|unweighted_case	pm2|pp2|pp3|ps3	fx|pm2|pp2|pp3	P	LP	VUS	P	P*	P*
c.172G>C	A58P	EF-hand 2	​	VUS	​	​	VUS	​	​	pm2|pp2|pp3|ppc	​	​	VUS	​	VUS	VUS	VUS
c.182A>G	N61S	EF-hand 2	​	VUS	​	​	​	​	​	​	​	​	VUS	​	VUS	VUS	VUS
c.188C>G	T63R	EF-hand 2	​	​	​	​	VUS	​	​	pm2|pp2|pp3|unweighted_case	​	​	VUS	​	VUS	VUS	VUS
c.192T>G	I64M	EF-hand 2	​	​	​	​	VUS	​	​	pm2|pp2|pp3|unweighted_case	​	​	VUS	​	VUS	VUS	VUS
c.203A>C	E68A	EF-hand 2	​	LP	​	​	LP	VUS	​	pm2|pp1|pp2|pp3|ppc	fx|pm2|pp2|pp3	​	LP	VUS	P*	P	P*
N/A	F69M	EF-hand 2	​	​	​	VUS	​	​	fx|pm2|pp2	​	​	VUS	​	​	VUS	VUS	VUS
N/A	F69A	EF-hand 2	​	​	​	VUS	​	​	fx|pm2|pp2	​	​	VUS	​	​	VUS	VUS	VUS
c.210G>T	L70F	EF-hand 2	VUS	​	​	​	​	​	​	​	​	VUS	​	​	VUS	VUS	VUS
c.214A>G	M72V	EF-hand 2	​	VUS	​	​	​	​	​	​	​	​	VUS	​	VUS	VUS	VUS
c.216G>T	M72I	EF-hand 2	​	​	​	VUS	​	​	pm2|pp2	​	​	VUS	​	​	VUS	VUS	VUS
c.216G>C	M72I	EF-hand 2	​	​	​	VUS	​	​	pm2|pp2	​	​	VUS	​	​	VUS	VUS	VUS
c.216G>A	M72I	EF-hand 2	​	​	​	VUS	​	​	pm2|pp2	​	​	VUS	​	​	VUS	VUS	VUS
c.247G>A	E83K	EF-hand 3	​	VUS	VUS	VUS	​	​	pm1|pm2|pp2|pp3|ppc	​	​	VUS	VUS	VUS	VUS	VUS	VUS
c.248A>G	E83G	EF-hand 3	​	​	​	​	LP	​	​	pm2|pm6|pp2|pp3|ppc	​	​	LP	​	P*	P	P*
c.260G>A	R87H	EF-hand 3	VUS	​	​	​	​	​	​	​	​	VUS	​	​	VUS	VUS	VUS
c.268T>C	F90L	EF-hand 3	LP	LP	​	P	VUS	​	fx|pm2|pp1m|pp2|pp3|ps3|ps4m	add_report|pm2|pp2|pp3|unweighted_case	​	P	LP	​	P	P*	P*
c.272G>T	R91L	EF-hand 3	​	VUS	​	​	​	​	​	​	​	​	VUS	​	VUS	VUS	VUS
N/A	F93A	EF-hand 3	​	​	​	VUS	​	​	fx|pm2|pp2	​	​	VUS	​	​	VUS	VUS	VUS
c.277T>C	F93L	EF-hand 3	​	​	​	VUS	​	​	fx|pm2|pp2|pp3	​	​	VUS	​	​	VUS	VUS	VUS
c.279T>G	F93L	EF-hand 3	​	​	​	VUS	​	​	fx|pm2|pp2|pp3	​	​	VUS	​	​	VUS	VUS	VUS
c.279T>A	F93L	EF-hand 3	​	​	​	VUS	​	​	fx|pm2|pp2|pp3	​	​	VUS	​	​	VUS	VUS	VUS
c.280G>C	D94H	EF-hand 3	VUS	​	​	​	​	​	​	​	​	VUS	​	​	VUS	VUS	VUS
c.281A>C	D94A	EF-hand 3	​	​	P	VUS	​	LP	fx|pm2|pp2|pp3	​	pm2|pp2|pp3|ps4m	VUS	​	P	P*	P*	P
c.286G>T	D96Y	EF-hand 3	P	P	​	​	​	​	​	​	​	P	P	​	P	P	P*
c.286G>C	D96H	EF-hand 3	​	​	P	LP	​	LP	fx|pm2|pm5|pp2|pp3	​	pm2|pp2|pp3|ps4m	LP	​	P	P*	P*	P
c.287A>G	D96G	EF-hand 3	VUS	​	​	​	LP	LP	​	pm2|pm6|pp2|pp3|ps4m	pm2|pm5|pp2|pp3|unweighted_case	VUS	LP	LP	P*	P	P
c.287A>T	D96V	EF-hand 3	​	P	​	LP	P	LP	fx|pm2|pp2|pp3|ps3	pm2|pm6|pp2|pp3|ps3|ps4m	fx|pm2|pp2|pp3|ps3	LP	P	LP	P*	P	P*
c.293A>G	N98S	EF-hand 3	P/LP	P	​	P	P	LP	fx|pm2|pm6|pp2|ps3|ps4m|unweighted_case	fx|pm2|pm6|pp2|pp3|ps3|ps4m	fx|pm2|pp2|ps3	P/LP	P	LP	P	P	P*
c.293A>C	N98T	EF-hand 3	​	​	​	LP	​	​	pm2|pm5|pp2|ppc	​	​	LP	​	​	P	P*	P*
c.293A>T	N98I	EF-hand 3	​	P	​	​	P	​	​	pm2|pm6|pp2|pp3|ps3|ps4m	​	​	P	​	P*	P	P*
c.294T>A	N98K	EF-hand 3	​	​	​	LP	​	​	pm2|pm6|pp2|ppc_het	​	​	LP	​	​	P	P*	P*
N/A	Y100T	EF-hand 3	​	​	​	VUS	​	​	fx|pm2|pp2	​	​	VUS	​	​	VUS	VUS	VUS
c.299A>G	Y100C	EF-hand 3	VUS	VUS	​	​	​	​	​	​	​	VUS	VUS	​	VUS	VUS	VUS
c.301A>G	I101V	EF-hand 3	VUS	​	​	​	​	​	​	​	​	VUS	​	​	VUS	VUS	VUS
c.307G>A	A103T	EF-hand 3	​	VUS	VUS	VUS	​	LP	pm2|pp2|pp3|unweighted_case	​	pm2|pm5|pp2|pp3|ppc	VUS	VUS	LP	P*	P*	P
c.308C>T	A103V	EF-hand 3	​	VUS	VUS	VUS	​	P	fx|pm2|pp2|pp3	​	fx|pm2|pp1m|pp2|pp3|ps3|ps4m	VUS	VUS	P	P*	P*	P
c.310G>C	E104Q	EF-hand 3	​	​	​	​	​	VUS	​	​	bp4|pm2|pp2	​	​	VUS	VUS	VUS	VUS
c.310G>A	A104T	EF-hand 3	​	VUS	VUS	​	​	​	​	​	​	​	VUS	VUS	VUS	VUS	VUS
c.311C>T	A104V	EF-hand 3	​	VUS	​	​	​	​	​	​	​	​	VUS	​	VUS	VUS	VUS
c.313G>A	E105K	EF-hand 3	LP	​	​	LP	​	​	pm2|pm5|pp2|pp3|ppc	​	​	LP	​	​	P	P*	P*
c.313G>C	E105Q	EF-hand 3	​	LP	​	​	​	​	​	​	​	​	LP	​	P*	P	P*
c.314A>C	E105A	EF-hand 3	​	​	​	P	​	​	fx|pm2|pm6|pp2|pp3|ps3|ps4m	​	​	P	​	​	P	P*	P*
c.319C>T	R107C	EF-hand 3	VUS	VUS	VUS	​	​	​	​	​	​	VUS	VUS	VUS	VUS	VUS	VUS
c.320G>A	R107H	EF-hand 3	​	​	VUS	​	​	​	​	​	​	​	​	VUS	VUS	VUS	VUS
c.322C>G	H108D	EF-hand 3	VUS	​	​	​	​	​	​	​	​	VUS	​	​	VUS	VUS	VUS
c.325G>A	V109I	EF-hand 3	​	​	VUS	​	​	​	​	​	​	​	​	VUS	VUS	VUS	VUS
N/A	M110A	EF-hand 3	​	​	​	VUS	​	​	fx|pm2|pp2	​	​	VUS	​	​	VUS	VUS	VUS
c.328A>C	M110L	EF-hand 3	VUS	​	​	​	​	​	​	​	​	VUS	​	​	VUS	VUS	VUS
c.328A>T	M110L	EF-hand 3	​	C	​	​	​	​	​	​	​	​	C	​	C	C	C
c.332C>T	T111M	EF-hand 3	​	​	VUS	​	​	​	​	​	​	​	​	VUS	VUS	VUS	VUS
c.340G>A	G114R	EF-hand 3	​	LP	​	​	LP	​	​	add_report|fx|pm2|pp2|ps3|unweighted_case	​	​	LP	​	P*	P	P*
c.340G>T	G114W	EF-hand 3	​	​	​	​	​	P	​	​	add_report|pm2|pm6|pp1|pp2|ppc|ps3	​	​	P	P*	P*	P
c.350T>C	L117S	EF-hand 4	​	VUS	​	​	​	​	​	​	​	​	VUS	​	VUS	VUS	VUS
c.355G>A	D119N	EF-hand 4	​	​	VUS	​	​	​	​	​	​	​	​	VUS	VUS	VUS	VUS
c.356A>T	D119V	EF-hand 4	​	​	​	​	VUS	​	​	pm2|pp2|pp3|unweighted_case	​	​	VUS	​	VUS	VUS	VUS
c.358G>A	E120K	EF-hand 4	VUS	​	​	​	​	​	​	​	​	VUS	​	​	VUS	VUS	VUS
c.365T>C	V122A	EF-hand 4	​	​	​	VUS	​	​	fx|pm2|pp2	​	​	VUS	​	​	VUS	VUS	VUS
c.367G>A	D123N	EF-hand 4	​	​	VUS	​	​	​	​	​	​	​	​	VUS	VUS	VUS	VUS
c.370G>A	E124K	EF-hand 4	​	​	VUS	​	​	​	​	​	​	​	​	VUS	VUS	VUS	VUS
c.379A>G	R127G	EF-hand 4	​	VUS	​	​	​	​	​	​	​	​	VUS	​	VUS	VUS	VUS
c.388G>C	D130H	EF-hand 4	​	​	VUS	​	​	​	​	​	​	​	​	VUS	VUS	VUS	VUS
c.388G>T	D130Y	EF-hand 4	​	​	​	​	LP	​	​	pm1|pm2|pp2|pp3|ppc	​	​	LP	​	P*	P	P*
c.388G>A	D130N	EF-hand 4	​	P	​	​	LP	LP	​	pm1|pm2|pp2|pp3|ppc	pm2|pm5|pp2|pp3|ppc	​	P	LP	P*	P	P*
c.389A>G	D130G	EF-hand 4	P/LP	LP	P	P	P	P	fx|pm2|pm6|pp2|pp3|ps3|ps4m	fx|pm1|pm2|pp2|pp3|ps3|ps4m|unweighted_case	pm2|pm6|pp1m|pp2|pp3|ps2|ps3|ps4m	P	P	P	P	P	P
c.389A>C	D130A	EF-hand 4	​	​	​	LP	​	​	pm2|pm5|pp2|pp3|ppc	​	​	LP	​	​	P	P*	P*
c.389A>T	D130V	EF-hand 4	​	​	​	LP	P	​	pm2|pm5|pp2|pp3|ppc	pm1|pm2|pm6|pp2|pp3|ps3|ps4m	​	LP	P	​	P*	P	P*
c.390C>G**	D130E	EF-hand 4	​	​	P	​	​	LP	​	​	pm2|pm5|pp2|pp3|ppc	​	​	P	P*	P*	P
c.392T>C	I131T	EF-hand 4	VUS	​	​	​	​	​	​	​	​	VUS	​	​	VUS	VUS	VUS
c.394G>A	D132N	EF-hand 4	P	​	VUS	VUS	​	​	fx|pm2|pm5|pp2	​	​	P	​	VUS	P	P*	P*
c.394G>C	D132H	EF-hand 4	​	​	​	LP	P	​	fx|pm2|pm5|pp2|pp3	pm1|pm2|pm6|pp2|pp3|ps3|ps4m	​	LP	P	​	P*	P	P*
c.394G>T	D132Y	EF-hand 4	​	P	​	​	LP	​	​	pm1|pm2|pp2|pp3|ppc	​	​	P	​	P*	P	P*
c.395A>G	D132G	EF-hand 4	P	P	LP	LP	LP	​	pm2|pm6|pp2|pp3|ppc_het	pm1|pm2|pp1|pp2|pp3|ps4m	​	LP	P	LP	P	P	P*
c.395A>T	D132V	EF-hand 4	P	​	VUS	P	​	​	fx|pm2|pm6|pp2|pp3|ps3|ps4m	​	​	P	​	VUS	P	P*	P*
c.396T>A	D132E	EF-hand 4	​	​	P	VUS	P	VUS	fx|pm2|pm5|pp2	pm1|pm2|pm6|pp2|pp3|ps3|ps4m	pm2|pp2|ppc	VUS	P	P	P*	P	P
c.396T>G	D132E	EF-hand 4	​	P	​	VUS	P	VUS	fx|pm2|pm5|pp2	pm1|pm2|pm6|pp2|pp3|ps3|ps4m	pm2|pp2|ppc	VUS	P	VUS	P*	P	P*
c.397G>A	G133S	EF-hand 4	​	LP	​	​	LP	​	​	pm1|pm2|pp2|pp3|ppc	​	​	LP	​	P*	P	P*
c.398G>C	G133A	EF-hand 4	VUS	​	​	​	​	​	​	​	​	VUS	​	​	VUS	VUS	VUS
c.398G>T	G133V	EF-hand 4	​	​	​	LP	​	​	pm1|pm2|pp2|pp3|ppc	​	​	LP	​	​	P	P*	P*
c.398G>A	G133E	EF-hand 4	LP	​	​	​	​	​	​	​	​	LP	​	​	P	P*	P*
c.400G>A	D134N	EF-hand 4	​	C	​	​	LP	​	​	pm1|pm2|pp1|pp2|pp3|ppc	​	​	LP	​	P*	P	P*
c.400G>C	D134H	EF-hand 4	​	P/LP	​	LP	P	​	fx|pm1|pm2|pp2|pp3	pm1|pm2|pm6|pp2|pp3|ps3|ps4m	​	LP	P	​	P*	P	P*
c.402C>G**	D134E	EF-hand 4	VUS	​	​	LP	​	​	pm1|pm2|pp2|pp3|ps4m	​	​	LP	​	​	P	P*	P*
c.402C>A**	D134E	EF-hand 4	​	​	​	LP	​	​	pm1|pm2|pp2|pp3|ps4m	​	​	LP	​	​	P	P*	P*
c.404G>A	G135E	EF-hand 4	​	​	​	LP	​	​	pm1|pm2|pp2|pp3|ppc	​	​	LP	​	​	LP	P*	P*
c.404G>T	G135V	EF-hand 4	​	​	​	​	LP	​	​	pm1|pm2|pp2|pp3|unweighted_case	​	​	LP	​	P*	P	P*
c.407A>C	Q136P	EF-hand 4	​	P/LP	​	P	P	​	fx|pm1|pm2|pp2|pp3|ps3	pm1|pm2|pm6|pp2|pp3|ps3|ps4m	​	P	P	​	P	P	P*
N/A	Q136M	EF-hand 4	​	​	​	VUS	​	​	fx|pm2|pm5|pp2	​	​	VUS	​	​	VUS	VUS	VUS
c.414C>A**	N138K	EF-hand 4	​	VUS	​	​	LP	​	​	pm1|pm2|pp2|pp3|ppc	​	​	LP	​	P*	P	P*
c.414C>G**	N138K	EF-hand 4	​	LP	​	​	LP	​	​	pm1|pm2|pp2|pp3|ppc	​	​	LP	​	P*	P	P*
c.414T>G**	N138K	EF-hand 4	​	​	​	​	​	P	​	​	pm2|pp1m|pp2|ps3|ps4m	​	​	P	P*	P*	P
c.419A>T	E140V	EF-hand 4	LP	​	​	​	​	​	​	​	​	LP	​	​	P	P*	P*
c.421G>A	E141K	EF-hand 4	​	​	P	​	LP	P	​	pm2|pm6|pp2|pp3|ps4m	pm2|pp2|pp3|ps2|ps3|ps4m	​	LP	P	P*	P*	P
c.422A>T	E141V	EF-hand 4	P	​	​	P	​	​	pm1|pm2|pm6|pp2|pp3|ps3|ps4m	​	​	P	​	​	P	P*	P*
c.422A>G	E141G	EF-hand 4	P	​	LP	P	LP	LP	fx|pm1|pm2|pm6|pp2|pp3|ps3|ps4m	pm2|pm5|pp2|pp3|ppc	pm2|pm5|pp2|pp3|ppc	P	LP	LP	P	P*	P*
c.423G>T**	E141D	EF-hand 4	​	​	​	​	LP	​	​	pm2|pm5|pp2|ppc	​	​	LP	​	P*	P	P*
c.423G>C**	E141D	EF-hand 4	​	LP	​	​	LP	​	​	pm2|pm5|pp2|ppc	​	​	LP	​	P*	P	P*
N/A	F142A	EF-hand 4	​	​	​	VUS	​	​	fx|pm2|pm5|pp2	​	​	VUS	​	​	VUS	VUS	VUS
c.424T>A	F142I	EF-hand 4	​	​	​	P	​	​	pm1|pm2|pp2|pp3|ppc_het|ps2	​	​	P	​	​	P	P*	P*
c.424T>C	F142L	EF-hand 4	P	​	​	P	LP	P	fx|pm1|pm2|pm6|pp2|pp3|ps3|ps4m	pm2|pp2|ps3	fx|pm2|pm6|pp2|ps3|ps4m	P	LP	P	P	P*	P
c.426T>G**	F142L	EF-hand 4	​	​	VUS	​	LP	P	​	pm2|pp2|ps3	fx|pm2|pm6|pp2|ps3|ps4m	​	LP	P	P*	P*	P
c.426T>A**	F142L	EF-hand 4	​	​	​	​	LP	P	​	pm2|pp2|ps3	fx|pm2|pm6|pp2|ps3|ps4m	​	P	P	P*	P*	P
c.426C>A**	F142L	EF-hand 4	P	​	​	P	​	​	fx|pm1|pm2|pm6|pp2|pp3|ps3|ps4m	​	​	P	​	​	P	P*	P*
c.426C>G**	F142L	EF-hand 4	P	​	​	P	​	​	fx|pm1|pm2|pm6|pp2|pp3|ps3|ps4m	​	​	P	​	​	P	P*	P*
c.434T>G	M145R	EF-hand 4	​	LP	​	​	​	​	​	​	​	​	LP	​	P*	P	P*
c.442G>A	A148T	EF-hand 4	​	​	VUS	​	​	​	​	​	​	​	​	VUS	VUS	VUS	VUS

“N/A” written in cDNA column if cDNA was not described in the published literature.

“*” listed in the Paralogue Annotation columns when “PA, criteria” were applied to the existing criteria for a variant to reach a P/LP (P) consensus call with an exact cDNA, match.

“**” variants with PA annotation applied where cDNA differences are present between genes with alignment on the amino acid residue change level. When P* is indicated for these variants, the code would be applied at the protein product level.

T30R/S: the c.89C>G change in *CALM1* and *CALM2* introduces an arginine, while in *CALM3*, it creates a serine.

Applied criteria definitions: PM1 (well-established functional domain or hot spot); PM2 (rare variant in gnomAD); PM5 (missense change at an amino acid residue where a different missense change is classified as P/LP); PM6 (assumed *de novo* variant without maternity and paternity confirmation); PS2 (*de novo* variant with confirmed maternity and paternity); PP1/PP1m (co-segregation of variant with disease in one or more families); PP2 (missense variant in a gene with low rate of benign missense variation); PP3 (multiple lines of computational evidence support a deleterious effect on the gene or gene product); PPC_het/PPC (heterozygous patient or presumed heterozygous patient with supported disease phenotype for gene of interest); PS4m (multiple patients with supported disease phenotype for gene of interest); Fx (functional data present with insufficient evidence for PS3 or BS3); PS3 (functional evidence in a well-established *in vitro* functional study supporting a damaging effect); Unweighted_case (patient identified has limited phenotypic information available, such as “sudden death” or whose phenotype does not have at least a moderate level of evidence associated with the gene); Add_report (patient previously published in another PMID); BP4 (multiple lines of computational evidence suggest no impact on gene or gene product).

### *CALM1*, *CALM2*, and *CALM3* variants from Mastermind

The Mastermind Genomic Intelligence Platform-based literature search identified 123 variants from published journal articles containing at least 1 variant in the CALM genes, comprising 59 *CALM1* variants, 40 *CALM2* variants, and 24 *CALM3* variants—123 variants in total (Table 1; Figure 2B). Within this dataset, *CALM1* comprised 32 VUS and 27 LP/P variants, *CALM2* 6 VUS and 34 LP/P variants, and *CALM3* 7 VUS and 17 P variants (Table 1).

### Combined ClinVar and Mastermind data

After merging data from ClinVar and Mastermind, the final dataset contained 126 unique cDNA variants coding for 110 unique protein variants (Table 2). The number of P/LP variants was 78 in Mastermind and 43 in ClinVar, whereas the number of VUS variants was 49 in ClinVar and 45 in Mastermind (Table 1). The overlap in variant classification between ClinVar and Mastermind databases was 20%, with 34 variants in common in both databases (33 P and 1 VUS; Table 1). If we compare variants that are missing from either database, Mastermind contained 45 *CALM1*, 20 *CALM2*, and 14 *CALM3* variants that were missing in ClinVar, respectively, while ClinVar contained 16 *CALM1*, 19 *CALM2*, and 15 *CALM3* variants that were missing in Mastermind. If we compare the variant classification of CALM genes in ClinVar and Mastermind, nine variants were classified as P/LP in Mastermind that were designated as VUS in ClinVar. They include two variants from *CALM1*: NM_006888.6:c.402C>G p.(Asp134Glu) and NM_006888.6:c.394G>A (p.Asp132Asn), three variants from *CALM2*: NM_001743.6:c.136G>A p.(Glu46Lys) and NM_001743:c.414C>A p.(Asn138Lys) and NM_001743:c.268T>C (p.Phe90Leu), and four variants from *CALM3*: NM_005184.4:c.307G>A p.(Ala103Thr), NM_005184.4:c.308C>T p.(Ala103Val),NM_005184.4:c.426T>G p.(Phe142Leu), and NM_005184.4:c.396T>A (p.Asp132Glu). Additionally, two *CALM2* variants were classified as having conflicting interpretations of pathogenicity (C) in the ClinVar dataset: NM_001743.6:c.400G>A p.(Asp134Asn), and NM_001743.6:c.328A>T p.(Met110Leu). Only one of these variants, NM_001743.6:c.400G>A p.(Asp134Asn), was resolved as P with the addition of Mastermind data, while the classification for the other variant, NM_001743.6:c.328A>T p.(Met110Leu), remained C (Table 1).

### Paralogue annotation

The data from ClinVar and Mastermind were combined into a single dataset, and conflicting classifications were resolved for *CALM1*, *CALM2*, and *CALM3* (Table 2) We applied the paralogue annotation (PA) conceptual code to each variant. Before PA, the dataset consisted of 126 unique cDNA and 111 protein variants across the three genes. With the application of PA as a strong criterion, the number of variants with evidence can then be multiplied by the three genes in the paralogue family, leading to a total of 378 variants to be classified across *CALM1*, *CALM2*, and *CALM3*. Before PA, the three genes had 84 VUS variants, 88 LP/P variants, and 1 conflicting classification (Table 2, “merged classification” columns).

After applying PA to the dataset, there was a 111.3% increase in P variants from 88 to 186—*CALM1* increased by 67.5%, *CALM2* increased by 82%, and *CALM3* increased by 195.2% (Figure 2C; Table 2.). For a description of what phenotype(s) are associated with ClinVar entries, please see Sheet 2 of Supplementary Table S1. For phenotypes extracted from the literature on a variant level basis, please see the “Combined_raw_data” sheet in Supplementary Table S1.

Conflicting classifications were found in 16 variants, 11 between ClinVar and Mastermind and five within either database, across different CALM genes (c.136G>A, c.161A>T, c.203A>C, c.268T>C, c.281A>C, c.287A>G, c.307G>A, c.308C>T, c.394G>A, c.395A>T, c.396T>A, c.396T>G, c.400G>A, c.402C>G, c.414C>A, and c.426T>G). During the process of merging classifications between ClinVar and Mastermind databases, five variants that contained VUS vs. LP/P conflicts within either database were resolved (c.268T>C, c.400G>A, c.402C>G, c.414C>A, and c.426T>G), leaving 11 variants with merged calls containing a conflict between CALM genes (Table 2). After PA, these classifications were resolved and marked as P* in Table 2.

### Mapping DNA variants to CaM protein

**FIGURE 3:**
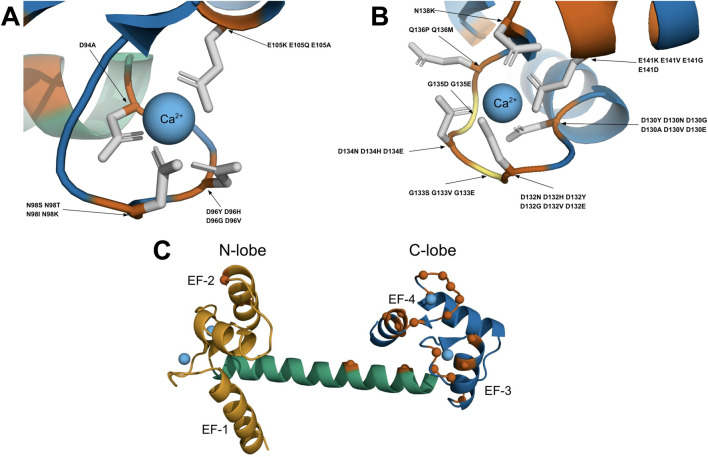
**(A)** 3D representation of C-lobe of the calmodulin (CaM) protein, highlighting the EF-hand 4 motif (labeled). Arrows point to the specific P variants reported in our dataset located at the specific locations colored in red. Key residues for protein function reported as P are shown with white side chains; dashed white lines indicate the distance (in angstroms, Å) between these residues and the bound calcium ion (Ca^2+^), potentially influencing CaM’s interaction with calcium and its downstream effects. **(B)** EF-hand 3 motif and variants located in this region. **(C)** Structure of the calmodulin protein in the holo form, a calcium-bound form essential for its function. Cyan spheres represent calcium ions (Ca^2+^). N-lobe (blue) and C-lobe (orange) are the functional domains of calmodulin, connected by the central tether (green). Notably, red spheres highlight the locations of pathogenic variants reported within the protein structure.

Calmodulin, encoded by *CALM1*, *CALM2*, and *CALM3*, is a highly conserved calcium-binding protein composed of two globular lobes (N- and C-terminal), each containing two EF-hand motifs: helix-loop-helix structural domains in which a 12-residue loop coordinates Ca^2+^ ions, enabling calcium-dependent conformational changes and target interaction. We sought to determine whether there were differences in the clustering of variants in different protein domains within our dataset, as reviewed by Jensen et al. (2018). Most of the pathogenic variants clustered in the C-lobe of the protein, while only two variants were positioned at the N-lobe: NP_001734.1:p.Glu46Lys and NP_001734.1:p.Asn54Ile (Figure 3C). Both these variants are associated with CPVT. On the other hand, the central tether region harbors pathogenic variants with three reported variants at two specific locations: NP_001734.1:p.Phe90Leu, NP_001734.1:p.Glu83Lys, and NP_001734.1:p.Glu83Gly. Notably, out of 57 P variants causing changes within the C-lobe, a significant portion of 47 variants affect residues that are directly involved in coordinating calcium ions (Ca^2+^). These variants directly affect the ability of the EF-hand 4 and 3 motifs to bind calcium (Figures 3A and B). Compared to EF-hand 3, which has 13 protein variants identified as P within the Ca^2+^ binding region, EF-hand 4 harbors a greater number of distinct protein P variants, with 26 in its Ca^2+^ binding region. It is interesting to note that 17.5% (ten variants) of both EF-hands do not directly affect calcium (Ca^2+^) binding.

## Discussion

Paralogue annotation between closely related proteins presents itself as a useful tool in variant annotation because the classification of a variant in one paralog can be applied to the paralogous variant in another gene (Li et al., 2023; Tarnovskaya et al., 2023; Walsh et al., 2014; Ware et al., 2012; Friedberg and Rhoads, 2001). The application of paralogue annotation in genomic diagnostics is significant because it decreases the likelihood of missed diagnosis. CALM genes are an example of a paralogous family, but many other examples exist, such as the DExD/H-box RNA helicase superfamily (Paine et al., 2019), the collagen gene family associated with Ehlers–Danlos syndrome, osteogenesis imperfecta (Ricard-Blum, 2011), dysplasias, the hemoglobin gene family, and the voltage-gated sodium channel family, beside others (Lal et al., 2020).

This study established a database of curated *CALM1, CALM2,* and *CALM3* variants from Mastermind and ClinVar and applied paralogue annotation to all variants in those genes. We demonstrated that the introduction of a literature-based search by Mastermind to the ClinVar dataset increased the number of reported pathogenic variants by 181% (from 43 to 78 variants) (Figure 2C). Additionally, paralogue annotation increased the number of pathogenic variants from 88 to 186 across all three genes (Figure 2C; Table 2). Previous studies have confirmed that paralogue annotation across calmodulin genes is a viable approach, as the p. Asp130Gly variant, initially detected in *CALM1*, was later found to induce LQTS when found in both *CALM2* and *CALM3* (Crotti et al., 2013; Reed et al., 2015; Boczek et al., 2016). Similarly, p. Phe142Leu was shown to cause LQTS in both *CALM2* and *CALM3* variants, further demonstrating that variants in one gene will have the same effect in another CALM gene (Crotti et al., 2013; Crotti et al., 2023). However, variants at the same protein site across different CALM genes can result in varying disease phenotypes, exemplified by the p. Asn98Ser-causing variant in *CALM1* associated with CPVT and in *CALM2* linked to LQTS (Maltret et al., 2021; Makita et al., 2014). The variant NM_006888.6:c.313G>A (p.Glu105Lys) in the *CALM1* gene can be used as an example of the PA application. This variant is reported in a patient in Crotti et al. (2019); Crotti et al. (2023) with LQTS like features but labeled as “atypical cardiac” by the authors. They classified the variant as LP (see the supplemental table in Crotti et al. (2019) for applied criteria). On review, we also curated this variant as LP (see Table 2 for applied ACMG criteria) and bucketed the phenotype described under LQTS. This cDNA change in *CALM2* and *CALM3* is absent in ClinVar and was not previously published on review. Using the PA application logic, if a moderate or strong code is applied for the paralogous relationship, this classifies those variants also as P/LP as they are also rare missense variants in regions of importance without expected splicing predictions (see Supplementary Table S2 for SpliceAI variant scoring using https://spliceailookup.broadinstitute.org/).

Paralogue annotation using the current ACMG classification codes could include the applications of PS1, PP2, PM1, or PM5 with some exclusion rules applied (Li et al., 2023; Richards et al., 2015; Brünger et al., 2023). Currently ACMG/AMP guidelines allow allelic evidence within the same gene: PS1 if the same amino acid or splice site region is altered or PM5 when a different substitution occurs at the same site. Paralogs were not originally included in the logic. The ClinGen RASopathy Expert Panel (VCEP) offers comparable gene groupings within this disease subset, identifying analogous functional residues and regions (Wilcox et al., 2025). This VCEP recommends the application of PS1_Strong for paralogous genes when “…the same amino acid change, either within the same gene or at an analogous position in a gene family member, has been previously classified as P.” ClinVar entry VCV000012610.40 is an example application of PA, PS1 code by this VCEP (National Center for Biotechnology Information, 2026). The Epilepsy Sodium Channel VCEP has also applied PA using PS1_Strong and PM5 for SCN1A, SCN2A, SCN3A, and SCN8A for variants with similar constraint scores; they note caution in the use of variants that impact splicing rather than impact at the amino acid/protein level (ClinGen Epilepsy Sodium Channel Variant Curation Expert Panel, 2025). This approach aims to attribute significance to a pathogenic variant in one gene based on its equivalent function in another gene within the same group. Proposed published guidance for variant interpretation of novel missense variants recommends using evidence from structurally equivalent positions, “meta-positions” within related protein domains from different genes, and “meta-domains” (Li et al., 2023). Cross-gene evidence can enhance the accuracy of both variant classification workflows and gene–disease relationship (GDR) assessments. Walsh et al. (2014) applied this approach to calmodulinopathy-causing genes, finding that individual assessments yielded varying levels of strength from moderate to limited association with CPVT. Similarly, Brünger et al. (2023) demonstrated for 36 genes that in code for paralogue proteins, the application of PS1 or PM5 criteria significantly reduces the number of VUSs. Hence, they suggest that para-PS1/PM5 should be applied in cases when PA is applicable and is an extension of the existing ACMG criteria for PS1 and PM5. The PA criterion code is applied to the variant/s under evaluation that are not already P but have a P/LP paralogous variant in the gene set. Nonetheless, future guidance on PA from ACMG to aid in the universal application of appropriate codes is needed for consistency of application across gene families and establishing a threshold of similarity required for the paralogue region on all appropriate levels of analysis (cDNA, protein, *in silico* predictions, tissue expression) for its application.

We also confirmed that most of the pathogenic variants are located at the C-lobe of the protein, with 57 out of 62 unique pathogenic variants found in this area, in line with previous findings that most pathogenic variants cluster there (Jensen et al., 2018). Furthermore, out of 57 pathogenic variants found in the C-lobe region, 47 are located within Ca^2+^ coordinating residues that are hypothesized to significantly decrease the affinity for Ca^2+^ (Figures 3A and B) (Gifford et al., 2007; Wang et al., 2020; Liu et al., 2019). All variants found in this region, except p. Glu140Val, are associated with the LQTS, which implies that variants in CaM that notably impact the affinity of the C-lobe for Ca^2+^ contribute to LQTS. Notably, previous research suggest a specific chelation order where EF-hand IV binds Ca^2+^ first, followed by EF-hand III, EF-hand II, and finally EF-hand I, facilitating precise allosteric regulation and positive cooperativity among the Ca^2+^ binding sites with the C-lobe sites (EF-hands III/IV) having higher-affinity for Ca^2+^ (Wang et al., 2020; Liu et al., 2019). With cooperation communication among EF-hands, the C-lobe influences the global CaM conformational state linked variants in EF-hand IV to disruptions of downstream activity of all the other hands. There is an increased presence of unique pathogenic variants in this small region, with 49% of unique pathogenic protein variants being located in EF-hand IV. On the other hand, only two variants were located at the N-lobe of the protein, p. Glu46Ly and p. Asn54Ile, and neither of them is found in Ca^2+^ coordinating residues.

A limitation of this study is that paralogue annotation of CALM genes does not account for varying tissue distribution and expression levels across different patient ages, which could potentially influence symptom severity and onset (Hussey et al., 2023). Additionally, the prevalence of predominantly *de novo* CaM mutations introduces the potential for mosaicism, where alterations during development lead to restricted expression in specific cell subsets or tissues (Wren et al., 2019). In addition, although the CALM genes encode identical calmodulin proteins, multiple lines of evidence suggest they are not fully functionally redundant (Christiansen et al., 2025; Bogdanov et al., 2025; Ohte et al., 2026). The preprint Christiansen et al. (2025) of clinical data from the International Calmodulinopathy Registry (ICalmR), population constraint analyses, and transcriptomic and translational profiling indicates that the genes differ in evolutionary constraint, expression levels, and contribution to the total calmodulin pool, with *CALM1* and *CALM2* accounting for the majority of cardiac calmodulin production and *CALM3* contributing substantially less.

In conclusion, we emphasize the importance of incorporating the paralogue annotation hypothesis code and literature-based searching into variant curation to increase the accuracy of epidemiological assessments. As the inheritance of CALM variants is typically *de novo*, obtaining enough criteria from one proband, especially as part of precision public health screening, may be exceedingly difficult. The hypothesized PA application derived from one variant to another in a homologous gene family may increase diagnostic rates, decrease VUSs, and could be implemented in clinical databases.

## Data Availability

Publicly available datasets were analyzed in this study. This data can be found at www.mastermind.genomenon.com.
