# Supplementary material for: Assessment of paralogue annotation for improving diagnostic accuracy in CALM1, CALM2, and CALM3 genes
Source: Front Genet. 2026 Jul 15;17:1761341. doi: 10.3389/fgene.2026.1761341 (PMC13413168; doi:10.3389/fgene.2026.1761341)
Supplement: Supplementary file 2 [file Table3.pdf]

Genomenon Variant Classification Standards

Preliminary Considerations

This document outlines the methodology used to classify sequence variants. The criteria listed below are based on Richards *et al.* “Standards and guidelines for the interpretation of sequence variants” (PMID: 25741868). Each of the points below are from the ACMG guidelines and the sub-bullets provide further explanation of how the criteria are applied.
The variant classifications are provisional and based on our curation of the published literature as well as additional data from population databases, computational algorithms, and intrinsic factors specific to the gene and/or condition. The date last reviewed is noted for each variant. Of note, while the classification guidelines are crucial in assessing a variant and ensure standardization, we recognize that:

Rigid criteria

While many aspects of a variant are fluid, the cutoffs for criteria are often rigid. For example, the minor allele frequency for a variant can span from absent to 50%; however, a benign population criterion, such as BS1, is applied at a specific cutoff. Variants just under that cutoff will not receive BS1 but may have a similar probability of being benign.

Discrepant classifications

Classifications may differ between experts. Discrepancies in classifications may be related to the criteria used and how the criteria are applied, the weight of different lines of evidence, internal and often unpublished data, and/or the external publications identified. Clinical laboratories or healthcare providers may have additional information on a variant (such as an unpublished patient or family) that may impact the overall classification.

We believe that all variant classifications should be transparent and supported with evidence. Users may independently assess the evidence, criteria applied, and the overall classification. We highly encourage submission of unpublished affected individuals with variants to a peer review journal, which enables all experts to utilize that data.

The classification is based on the currently available evidence. New evidence or changes to how classification criteria are applied may result in a different classification. **Anyone with questions about the evidence, criteria applied, or the overall classification or anyone with knowledge of published data that is not present in the interpretation is welcome to contact us.**

Expert Group Modifications

In some cases, modifications to the application of criteria or the strength applied to the criteria are recommended by expert groups. These modifications may be recommended for the criteria as a whole or based on a specific gene or disease. We regularly review publications and the modifications suggested by these expert groups and independently assess each modification for inclusion into our standards.

Variants with conflicting evidence

The classification "conflict" is used for variants with sufficient evidence to deem a variant pathogenic and benign. For these variants, there is enough pathogenic criteria to classify as pathogenic/likely pathogenic and enough benign criteria to classify as benign/likely benign. The "conflict" classification differentiates these variants from those with insufficient evidence, which are classified as "variant of uncertain significance".

Pseudogenes

Pseudogenes can pose a challenge for variant classification. For regions of high homology, population criteria (BA1, BS1, and PM2) are not applied. Clinical evidence identified for variants in regions of high homology is curated under the appropriate ACMG criteria. This clinical evidence may not be associated with the functional gene but instead be present in the pseudogene; however, the curation of this clinical evidence is meant to emphasize the potential criteria that may be applied.

Criteria

The criteria listed below are divided into categories: population, computational, intrinsic, clinical, and functional based criteria.

Population

- Population based criteria assesses the variant's prevalence in affected and unaffected individuals. The standard for determining the prevalence of a variant in the general population is large genomic databases (for example: gnomAD). Of note, at the time the ACMG guidelines were published, gnomAD had not yet been released; however, gnomAD is currently the largest and most comprehensive dataset and it contains all the data of the other major sequencing project mentioned in ACMG criteria, including Exome Sequencing Project, 1000 Genomes Project, and Exome Aggregation Consortium (PMID: 31479589).

Benign population categories

- **BA1 - Very common** - Allele frequency is >5% in Exome Sequencing Project, 1000 Genomes Project, or Exome Aggregation Consortium.
    - This criterion is applied based on population data in gnomAD.

- **BS1 - Too common** - Allele frequency is greater than expected for disorder.
    - This criterion is applied based on population data in gnomAD when the allele frequency is greater than a cutoff for the condition. The default cutoff is 1%; however, this cutoff may be raised or lowered based on factors including the prevalence of the disorder, the inheritance pattern, the frequency of the most prevalent pathogenic variant(s), and expert group recommendations.

<u>Pathogenic population categories</u>

- **PM2 – Rare** - Absent from controls (or at extremely low frequency if recessive) in Exome Sequencing Project, 1000 Genomes Project, or Exome Aggregation Consortium.
    - This criterion is applied based on absence of the variant in gnomAD or presence at an allele frequency less than 0.05% in all populations.
        - Of note, two subpopulations, Ashkenazi Jewish and Finnish, have been identified to have overrepresentation of rare pathogenic alleles in the gnomAD dataset (PMID: 30311383). Therefore, if an allele is >0.05% in one or both subpopulations but no other subpopulation, then PM2 is still applied.

Intrinsic

<u>Benign intrinsic categories</u>

- **BP1 - Missense not primary mechanism** - Missense variant in a gene for which primarily truncating variants are known to cause disease.
    - This criterion is applied for all missense variants in genes where the mechanism of disease is loss of function and null variants are exclusively or almost exclusively pathogenic.

- **BP3 - In-frame deletion/duplication in repetitive region** - In-frame deletions/insertions in a repetitive region without a known function.
    - This criterion is applied for an inframe deletion/duplication in a repetitive region of a gene for which the region is not in an established functional domain based on information from public protein databases or in peer reviewed publications

- **BP7 - Synonymous** - A synonymous (silent) variant for which splicing prediction algorithms predict no impact to the splice consensus sequence nor the creation of a new splice site AND the nucleotide is not highly conserved.
    - This criterion is applied for synonymous variants in which splicing algorithms do not predict an impact on splicing.

<u>Pathogenic intrinsic categories</u>

- **PVS1 – Null variant** - null variant (nonsense, frameshift, canonical ±1 or 2 splice sites, initiation codon, single or multi exon deletion) in a gene where LOF is a known mechanism of disease.
    - This criterion is applied to null variants in genes where loss of function is a known or likely mechanism of disease.
        - Of note, caution should be used when classifying truncating variants in the last exon or within approximately 50 nucleotides of the final exon/intron boundary in the second to last exon of a gene (PMID: 30192042). These variants may not be subject to nonsense mediated decay and, therefore, may not result in a null variant. Truncating variants in this region are flagged to note caution in considering PVS1.

- **PS1 – Same amino acid change** - Same amino acid change as a previously established pathogenic variant regardless of nucleotide change.
    - Protein coding variants in Mastermind are represented based on the protein variant rather than the nucleotide variant. As nucleotide variants are not represented independently, PS1 would not be applied for a protein coding variant. PS1 may be utilized for intronic variants.

- **PM1 - Domain or hotspot** - Located in a mutational hot spot and/or critical and well-established functional domain (e.g., active site of an enzyme) without benign variation.
    - This criterion is applied to variants that are in an established functional domain based on information from public protein databases or in peer reviewed publications. This criterion is applied to variants in a hotspot or hotspot regions.

- **PM4 – In-frame deletion/duplication** - Protein length changes as a result of in-frame deletions/insertions in a nonrepeat region or stop-loss variants.
    - This criterion is applied to in-frame deletion or duplication outside of repetitive regions. This criterion is also applied to splicing variants where the reported impact of the variant is a deletion of a single small in-frame exon without any known functional domains or insertion of several additional residue in-frame.

- **PM5 - Different missense change** - Novel missense change at an amino acid residue where a different missense change determined to be pathogenic has been seen before.
    - This criterion is applied to missense variants in which a different missense variant at the same residue is classified as pathogenic or likely pathogenic.

- **PP2 – Missense typically pathogenic** - Missense variant in a gene that has a low rate of benign missense variation and in which missense variants are a common mechanism of disease.
    - This criterion is applied for all missense variants in genes where missense variants are a common mechanism of disease and where benign missense variation is rare.

Computational

Benign computational categories

- **BP4 – Computationally inert** - Multiple lines of computational evidence suggest no impact on gene or gene product (conservation, evolutionary, splicing impact, etc.).
    - This criterion is applied for variants in which neither the protein prediction model (REVEL) nor the splicing algorithm (dbscSNV) predict an impact.

Pathogenic computational categories

- **PP3 - Predicted damaging** - Multiple lines of computational evidence support a deleterious effect on the gene or gene product (conservation, evolutionary, splicing impact, etc.).
    - This criterion is applied for variants in which either the protein prediction model (REVEL) and/or the splicing algorithm (dbscSNV) predict an impact.

Clinical

Benign clinical categories

- **BS2 - Healthy individual** - Observed in a healthy adult individual for a recessive (homozygous), dominant (heterozygous), or X-linked (hemizygous) disorder, with full penetrance expected at an early age.
    - This criterion is applied when a variant is identified in literature in a healthy adult individual for a recessive (homozygous), dominant (heterozygous), or X-linked (hemizygous) condition. This criterion is only applied when the associated condition has complete penetrance at an early age and in which the condition does not have significant phenotypic variability to where a mildly affected individual may be labeled as unaffected.

- **BS4 - Lack of segregation** - Lack of segregation in affected members of a family.
    - This criterion is applied when a variant is identified in literature where one or more affected members of the family are found to not carry the variant.

- **BP2 - In cis with pathogenic variant** - Observed in trans with a pathogenic variant for a fully penetrant dominant gene/disorder or observed in cis with a pathogenic variant in any inheritance pattern.
    - This criterion is applied when a variant is identified in literature either in trans with a variant classified as pathogenic/likely pathogenic for a fully penetrant dominant condition or in *cis* with a variant classified as pathogenic/likely pathogenic.

- **BP5 – Alternate molecular basis** - Variant found in a case with an alternate molecular basis for disease.
    - This criterion is applied when a variant is identified in literature in an affected individual who is identified to have a pathogenic variant that explains their condition.

<u>Pathogenic clinical categories</u>

- **PS2 - *De novo*** - *De novo* (both maternity and paternity confirmed) in a patient with the disease and no family history.
    - This criterion is applied when a variant is identified in literature in an affected individual and is found to be *de novo* with evidence that the study assessed and confirmed both maternity and paternity.

- **PS4 - Highly prevalent in cases** - The prevalence of the variant in affected individuals is significantly increased compared with the prevalence in controls.
    - This criterion is applied for large case control studies in which a variant is found to be identified in cases significantly more often than in controls.
    - PS4M - a criteria used for literature-based application in multiple affected individuals and is applied as a moderate level of evidence of pathogenicity. This criterion is applied when a variant is identified in two more unrelated affected individuals, either in the same publication or separate publications.
    - In situations where PS4M and PPC are applied to the same variant, only PS4M is counted for the purpose of classification.
        - Of note, curation of medical literature involves reviewing publications with affected individuals to determine whether they have previously been published. Patients that are reported in more than one publication should only be represented once within the curation. However, in rare situations a single patient may be counted twice from two separate publications (e.g., there is insufficient information in a publication to identify a patient has been previously published). This rare event may lead to PS4M being applied if that one is the only reported affected individual with the variant.

- **PM3 - In trans with pathogenic variant** - For recessive disorders, detected in trans with a pathogenic variant.

- This criterion is applied when a variant is identified in literature in an affected individual in trans with a variant classified as pathogenic/likely pathogenic for a recessive condition.

- **PM6 – Assumed *de novo*** - Assumed *de novo*, but without confirmation of paternity and maternity.
    - This criterion is applied when a variant is identified in literature in an affected individual and is found to be de novo; however, without evidence that the study assessed and/or confirmed both maternity and paternity.

- **PP1 - Segregation with disease** - Cosegregation with disease in multiple affected family members in a gene definitively known to cause the disease.
    - This criterion is applied when a variant is identified in literature where at least two affected family members are tested and have the variant, and no affected family members are found to not carry the variant. This criterion is applied when segregation is noted to occur in a single family.

- **PP1M - Segregation with disease - multiple families** - Cosegregation with disease in multiple affected family members in a gene definitively known to cause the disease.
    - This criterion is applied when a variant is identified in literature where at least two affected family members in at least two families are tested and have the variant, and, in the case of rare disease, no affected family members are found to not carry the variant.
    - This criterion is applied when segregation is identified in two or more unrelated families, either in the same publication or separate publications.
    - In situations where PP1M and PP1 are applied to the same variant, only PP1M is counted for the purpose of classification.

- **PP4 - Family history** - Patient's phenotype or family history is highly specific for a disease with a single genetic etiology.
    - This criterion is not used for patients identified in literature.
    - PPC – a criteria used for literature-based application in affected individuals. This criterion is applied when a variant is identified in a single affected individual in one publication. A modifier is used to indicate the state of the variant (e.g., PPC_HET, PPC_HOMO, and PPC_COMPOUND for heterozygous, homozygous, and compound heterozygous, respectively).
    - As noted above, when a variant is published in a single patient in more than one study and the patients are unique, then the criteria PS4M is applied for the purpose of classification rather than PPC. However, in the interpretation view, the individual publications will be listed under the appropriate PPC header.

Unweighted clinical categories

- **ADD REPORT**
    - This category is used when a patient or cohort of patients is identified to have been previously reported (an additional report). This category is considered unweighted for the purpose of classification.

- **UNWEIGHTED CASE**
    - This category is used in situations where an affected patient is identified; however, the information presented in the article is insufficient to determine whether the case should be counted towards pathogenicity. Examples of the use of this category include: an individual who is mildly affected in which their published phenotype is insufficient or potentially insufficient for a clinical diagnosis or an affected individual who was identified to have multiple variants and there is insufficient information to determine which variant is potentially causative.

- **REVIEW**
    - This category is used to identify references that are review articles and/or articles that present no new information about the variant that would be useful in classification.

Functional

Benign functional category

- **BS3 - No functional impact** - Well-established *in vitro* or *in vivo* functional studies show no damaging effect on protein function or splicing.
    - This criterion is applied when a variant is identified in literature to have no damaging effect on the protein or gene in an assay intended to assess the mechanism of disease directly or indirectly.

Pathogenic functional category

- **PS3 - Functionally consequential** - Well-established *in vitro* or *in vivo* functional studies supportive of a damaging effect on the gene or gene product.
    - This criterion is applied when a variant is identified in literature to have a damaging effect on the protein or gene in an assay intended to assess the mechanism of disease directly or indirectly.

Unweighted functional category

- **FX**

- This category is used to identify functional data which is insufficient to apply either PS3 or BS3.
- The category may be applied because the assay does not directly assess a mechanism of disease or the result of the study shows a moderate impairment

External sources

Benign external sources category

- **BP6 - Reputable source – benign** - Reputable source recently reports variant as benign, but the evidence is not available to the laboratory to perform an independent evaluation.
    - Classification should be based on primary data and not expert opinion without access to the primary data and use of this criterion may result in double counting of the same set of data and an error in classification (PMID: 29543229). Therefore, this criterion is not used in our classifications.

Pathogenic external sources category

- **PP5 - Reputable source – pathogenic** - Reputable source recently reports variant as pathogenic, but the evidence is not available to the laboratory to perform an independent evaluation.
    - Classification should be based on primary data and not expert opinion without access to the primary data and use of this criterion may result in double counting of the same set of data and an error in classification (PMID: 29543229). Therefore, this criterion is not used in our classifications.

Calculation of the Provisional Classification

The provisional classification is based on Richards *et al.* “Standards and guidelines for the interpretation of sequence variants” (PMID: 25741868), with the exception that one strong benign criterion is sufficient to classify as likely benign (PMID: 29300386).

Evidence identified from variants listed in the full text of peer reviewed publications are included in our curations. For variants that are very well published, evidence may be curated until there is sufficient data to suggest a classification. Supplemental data is not routinely curated.

References

Abou Tayoun AN, *et al*. ClinGen Sequence Variant Interpretation Working Group (ClinGen SVI). Recommendations for interpreting the loss of function PVS1 ACMG/AMP variant criterion. Hum Mutat. 2018 Nov;39(11):1517-1524. doi: 10.1002/humu.23626. (PMID: 30192042).

Adzhubei IA, *et al*. A method and server for predicting damaging missense mutations. Nat Methods. 2010 Apr;7(4):248-9. doi: 10.1038/nmeth0410-248. (PMID: 20354512).

Biesecker LG and Harrison SM. ClinGen Sequence Variant Interpretation Working Group. The ACMG/AMP reputable source criteria for the interpretation of sequence variants. Genet Med. 2018 Dec;20(12):1687-1688. doi: 10.1038/gim.2018.42. (PMID: 29543229).

Ghosh R, *et al*. ClinGen Sequence Variant Interpretation Working Group. Updated recommendation for the benign stand-alone ACMG/AMP criterion. Hum Mutat. 2018 Nov;39(11):1525-1530. doi: 10.1002/humu.23642. (PMID: 30311383).

Harrison SM, *et al*. Overview of Specifications to the ACMG/AMP Variant Interpretation Guidelines. Curr Protoc Hum Genet. 2019 Sep;103(1):e93. doi: 10.1002/cphg.93. (PMID: 31479589).

Jian X, *et al*. In silico prediction of splice-altering single nucleotide variants in the human genome. Nucleic Acids Res. 2014 Dec 16;42(22):13534-44. doi: 10.1093/nar/gku1206. (PMID: 25416802).

Karczewski KJ, *et al*. The mutational constraint spectrum quantified from variation in 141,456 humans. Nature. 2020 May;581(7809):434-443. doi: 10.1038/s41586-020-2308-7. Epub 2020 May 27. Erratum in: Nature. 2021 Feb;590(7846):E53. (PMID: 32461654).

Liu X, *et al*. dbNSFP: a lightweight database of human nonsynonymous SNPs and their functional predictions. Hum Mutat. 2011 Aug;32(8):894-9. doi: 10.1002/humu.21517. (PMID: 21520341).

Liu X, *et al*. dbNSFP v4: a comprehensive database of transcript-specific functional predictions and annotations for human nonsynonymous and splice-site SNVs. Genome Med. 2020 Dec 2;12(1):103. doi: 10.1186/s13073-020-00803-9. (PMID: 33261662).

Richards S, *et al*. Standards and guidelines for the interpretation of sequence variants: a joint consensus recommendation of the American College of Medical Genetics and Genomics and the Association for Molecular Pathology. Genet Med. 2015 May;17(5):405-24. doi: 10.1038/gim.2015.30. Epub 2015 Mar 5. (PMID: 25741868).

Schwarz JM, *et al*. MutationTaster2: mutation prediction for the deep-sequencing age. Nat Methods. 2014 Apr;11(4):361-2. doi: 10.1038/nmeth.2890. (PMID: 24681721).

Tavtigian SV *et al*. Modeling the ACMG/AMP variant classification guidelines as a Bayesian classification framework. Genet Med. 2018 Sep;20(9):1054-1060. doi: 10.1038/gim.2017.210. Epub 2018 Jan 4. (PMID: 29300386).

Vaser R, *et al*. SIFT missense predictions for genomes. Nat Protoc. 2016 Jan;11(1):1-9. doi: 10.1038/nprot.2015.123. Epub 2015 Dec 3. (PMID: 26633127).
