# Supplementary material for: Assessment of paralogue annotation for improving diagnostic accuracy in CALM1, CALM2, and CALM3 genes
Source: Front Genet. 2026 Jul 15;17:1761341. doi: 10.3389/fgene.2026.1761341 (PMC13413168; doi:10.3389/fgene.2026.1761341)
Supplement: Supplementary file 4 [file DataSheet1.docx]

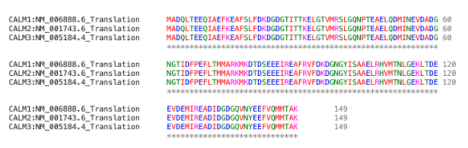
**Supplemental Figure 1.** Clustal Omega Multiple sequence alignment of calmodulin translated protein products


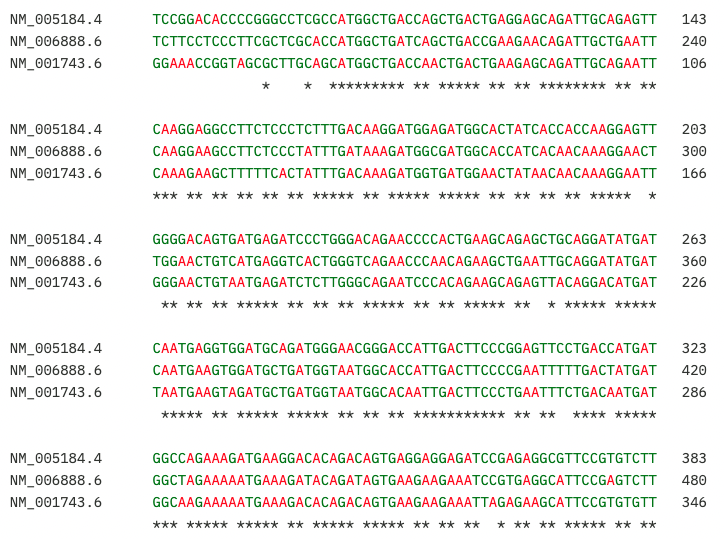


**Supplemental Figure 2.** Clustal Omega Multiple sequence alignment of calmodulin cDNA within the coding region.
